# Cervical pedicle screw fixation with the Tianji orthopedic surgical robot

**DOI:** 10.1186/s13018-024-05325-3

**Published:** 2025-02-04

**Authors:** Hao Shen, Jinlong Zhou, Lipeng Yu

**Affiliations:** The First Affiliated Hospital with Nanjing Medical University, Nanjing, China

**Keywords:** Cervical pedicle screw, CPS, Orthopedic surgical robot, Neo scale, Tianji

## Abstract

**Objective:**

To compare the accuracy and safety of implanting cervical pedicle screws (CPS) between orthopedic surgical robot-assisted technique and traditional fluoroscopy-assisted free-hand technique.

**Methods:**

Retrospective analysis of 95 patients treated with posterior cervical spinal surgery using either Tianji orthopedic surgical robot-assisted or traditional fluoroscopy-assisted free-hand pedicle screw implantation technology from March 2021 to March 2024, including 44 cases in the orthopedic surgical robot group and 51 cases in the traditional fluoroscopy group.

**Results:**

Compared with the traditional fluoroscopy group, the orthopedic surgical robot group had better accuracy in screw implantation that is, a higher acceptable rate of screws (*p* = 0.0083). In addition, compared with the traditional fluoroscopy group, postoperative hospital stay was shorter in the orthopedic surgical robot group (*p* = 0.0447), but operation duration was longer (*p* = 0.0038). There was no significant difference in intraoperative blood loss between groups (*p* = 0.0872). There were 2 cases of cerebrospinal fluid leakage and 1 case of decreased left handgrip strength in the traditional fluoroscopy group, while only 1 case of cerebrospinal fluid leakage occurred in the orthopedic surgical robot group.

**Conclusions:**

In this retrospective study, the accuracy of spine surgery with CPS implantation assisted by orthopedic surgical robot is often superior to that of spine surgery using traditional fluoroscopy-guided CPS implantation technique, while maintaining comparable safety.

## Introduction

Cervical spinal disease is considered one of the leading causes of human disability. The preferred treatment for cervical spinal disease is conservative therapy, and surgery is considered when conservative therapy is ineffective or when it seriously impairs quality of life [1]. The placement of screws, including pedicle screws and lateral mass screws, is of significance to maintain the stability of the cervical spine [2]. However, compared to lateral mass screws, pedicle screws have higher technical requirements and greater risks, so their application is limited. The technique of CPS fixation was first studied by Abumi et al. in 1994 and has been widely applied in clinical practice due to its excellent biomechanical stability [3]. Compared to the thoracic and lumbar vertebrae, the cervical vertebrae have smaller diameter of pedicles [4], which makes it very difficult to implant pedicle screws. Besides, the cervical spine is in close proximity to many important tissues, including the spinal cord, vertebral artery, and nerve roots. Therefore, improper placement of screws may lead to decreased stability, even serious neurological or vascular complications [5]. Traditional fluoroscopy-assisted pedicle screw placement is judged through intraoperative two-dimensional and preoperative CT reconstructed images to determine the entry point and direction of the screws [6]. The surgeon’s spatial judgment and proficiency in operation have a significant impact on the accuracy of screw placement, resulting in differences in accuracy among different surgeons. Especially when placing pedicle screws in the cervical spine, it is more difficult and increases the possibility of poor prognosis, making high technical requirements for pedicle screw placement, and a long learning curve under fluoroscopy-guided screw placement.

In recent years, the emergence of orthopedic surgical robots is expected to solve this problem. In 1992, the ROBODOC was the first robot to be employed for orthopedics by boring a hole in the femoral head, allowing surgeons to optimize prosthesis size for total hip arthroplasty [7]. After years of improvement, the Tianji Robot (TINAVI Medical Technologies Co., Ltd., Beijing, China) was approved for clinical use by the China Food and Drug Administration in 2016, and achieved the world’s first robot-assisted upper cervical spine surgery [8–11]. With the assistance of orthopedic surgical robot, the surgeon only needs to plan the screw path on the image, control the robot arm to run to the specified position, and the sleeve at the end of the robot arm can accurately indicate the screw point and the direction of the screw path [12]. 

This study retrospectively compared the accuracy of screw implantation in cervical spine surgery using this robot-assisted technique versus conventional fluoroscopy-assisted free-hand technique. Intraoperative blood loss, duration of surgery, and postoperative hospital stay length were also compared. The safety of pedicle screw implantation was evaluated based on postoperative complications.

## Materials and methods

### Study design and participants

In this case-control study, patients were recruited and managed at Jiangsu Provincial People’s Hospital (JSPH). The study commenced two years after the introduction of the Tianji Robot for spinal surgery at JSPH in order to avoid the learning curve [13]. Inclusion criteria were (1) newly diagnosed cervical spinal disease requiring pedicle screw fixation; (2) patients undergoing posterior cervical internal fixation with CPS assisted by orthopedic surgical robot or traditional fluoroscopy. Exclusion criteria were the presence of (1) severe osteoporosis; (2) old fractures; (3) severe pedicle deformity; (4) cervical pedicle with diameter smaller than the screw diameter (3.5 mm); (5) preoperative CTA examination indicating that unilateral vertebral artery stenosis or atresia and (6) severe systemic disease or coagulation disorder. Other types of screws, such as lateral mass screws, were excluded.

### Participants characteristics

From March 2021 to March 2024, altogether 95 patients were treated with posterior cervical spinal surgery using either Tianji orthopedic surgical robot-assisted or traditional fluoroscopy-assisted pedicle screw implantation technology, including 44 cases in the orthopedic surgical robot group and 51 cases in the traditional fluoroscopy group. The orthopedic surgical robot group consisted of 30 males, 14 females, aged from 23 to 82 years old, with a median age of 57 years. There were 24 cases of cervical fracture with cervical dislocation or not, 13 cases of cervical spinal stenosis, 1 case of benign intraspinal tumor, 3 cases of cervical malignancy, 1 case of cervical kyphosis, 1 case of congenital cervical deformity, and 1 case of basilar invagination in this group. The traditional fluoroscopy group consisted of 37 males, 14 females, aged from 40 to 81 years old, with a median age of 59 years. In this group, there were 22 cases of cervical fracture with cervical dislocation or not, 19 cases of cervical spinal stenosis, 5 case of benign intraspinal tumor, 5 cases of cervical malignancy.

### Interventions

The doctors of CPS implantation were senior spine surgeons with many years of experience in implantation of pedicle screws manually and were skilled in using robots for pedicle screw fixation. All participants received the pedicle screw fixation assisted by orthopedic surgical robot or conventional fluoroscopy.

### Orthopedic surgical robot-assisted cervical spinal surgery

**Fig. 1:**
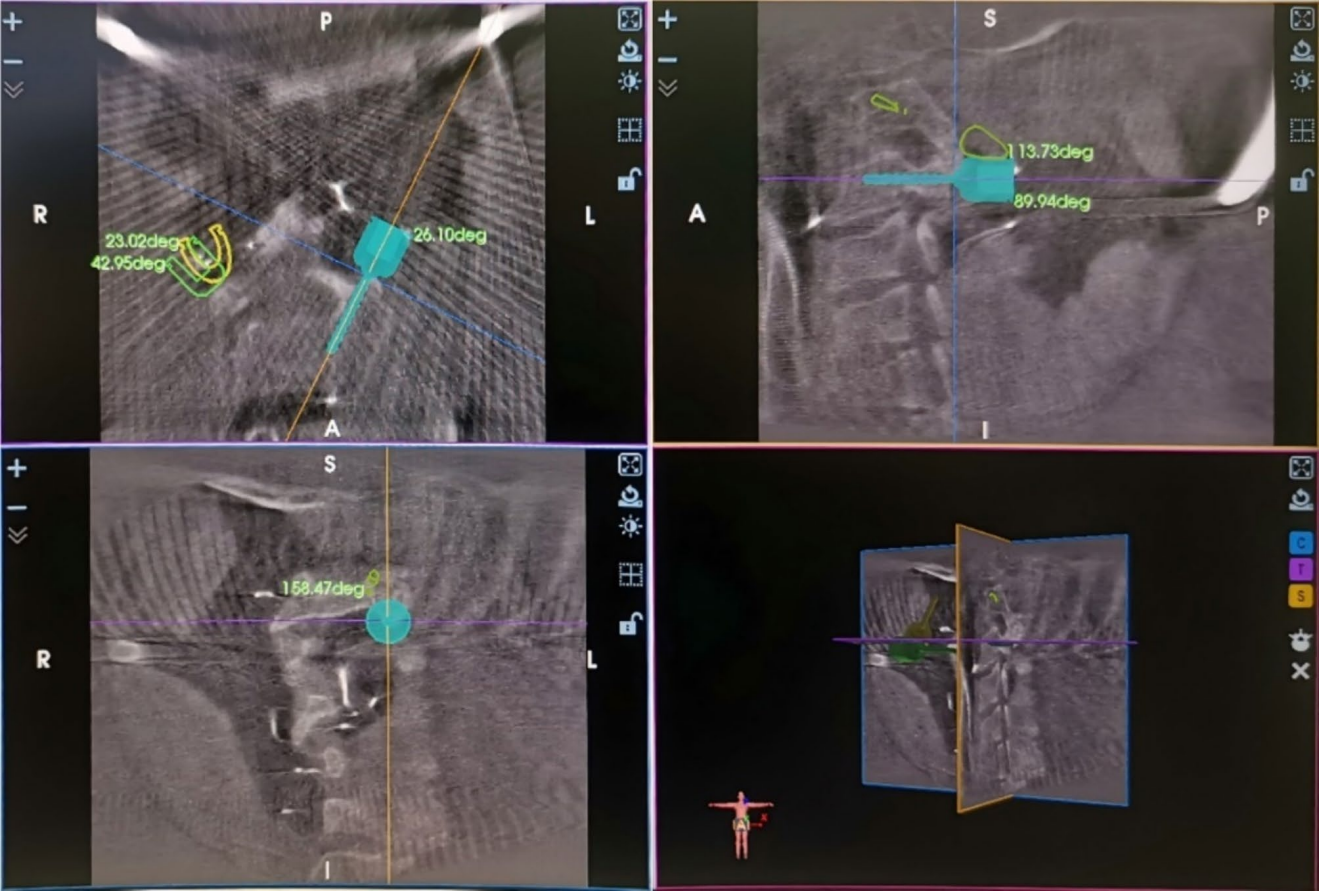
Intraoperative localization image of the orthopedic surgical robot group A patient with cervical dislocation underwent reduction and pedicle screw fixation using TINAVI robotic navigation system to implant CPS at C2 and C3. The figure showed the planning of the screw trajectory during the operation

The patient is placed in a prone position on a Jackson table after general anesthesia, with the head secured by a Mayfield frame, and the area is sterilized and covered with a sterile sheet. A longitudinal midline incision is made at the back of the neck, and the target segment’s spinous processes, pedicles, and facet joints are exposed by subperiosteal dissection. The human navigation recognition framework is fixed to the distal spinal process or the proximal head frame. The orthopedic surgical robot system (TINAVI Medical Technologies Co., Ltd., Beijing, China) with a 3D “C”-arm x-ray machine (Siemens Medical Solutions, Erlangen, Germany) is used for standard operating procedures to perform 3D scanning. After the scan is completed, the pedicle trajectory is planned on the robot workstation and the mechanical arm is controlled to the target area. A guide needle sleeve is inserted to confirm the pedicle entry point, and the cortical bone is ground off using a grinding drill at the entry point. A 1.2 mm diameter pedicle guide needle is drilled into the bone using an electric drill along the sleeve, and the procedure is repeated with the mechanical arm to insert all guide needles. After confirming the position of the guide needle through fluoroscopy, a 2.7 mm hollow drill is used to enlarge the hole along the guide needle direction, and the guide needles are removed and tapped. A round-headed probe is used to check the screw hole, and the 3.5 mm diameter pedicle screw is tightened(Figure 1).

Conventional Fluoroscopy-assisted Cervical Spinal Surgery: The patient is placed in a prone position after general anesthesia, with the head secured by a Mayfield frame, and the area is sterilized and covered with a sterile sheet. A longitudinal midline incision is made at the back of the neck, and the target segment’s spinous processes, pedicles, and facet joints are exposed by subperiosteal dissection. Refer to Abumi pedicle implantation method to confirm the pedicle screw insertion point [13]. Use a grinding drill to remove the cortical bone at the insertion point, and drill and tap manually. Use a round probe to check the screw hole without error, and insert the pedicle screw manually.

### Definitions of outcomes

Sociodemographic data assessed at baseline (preoperatively) included age, sex, and BMI. The postoperative CT scans were acquired in 1 week after surgery. The primary outcome was the accuracy of the screw implantation, which was evaluated according to Neo scale assessed by the postoperative CT [14]. Intraoperative blood loss, duration of surgery, and postoperative hospital stay length were also compared. The safety of pedicle screw implantation was evaluated based on postoperative complications, including cerebrospinal fluid leakage, spinal cord injury, nerve root injury, incidence of infection, vertebral artery injury.

### Neo scale

Grade 0: screw completely within bone.

Grade 1: cortical breach of < 2 mm.

Grade 2: cortical breach of ≥ 2 mm and < 4 mm.

Grade 3: cortical breach of ≥ 4 mm.

### Statistical analysis

All statistical analyses were performed using the SPSS Statistics version 25.0 software (IBM, Armonk, NY). All tests were two-tailed with an α of 0.05.

## Results

From March 2021 to March 2024, altogether 95 patients underwent posterior cervical spinal surgery (44 cases in the orthopedic surgical robot group and 51 cases in the traditional fluoroscopy group). The baseline sociodemographic characteristics and diagnosis were balanced between the two groups (Tables 1 and 2). The mean age of the overall study population was 57 years, 29.47% were women, and the average body mass index was 23.22 kg/m^2^. Regarding to the diagnosis, 48.42% of the overall study population were cervical fracture, 33.68% were cervical spinal stenosis, and 17.89% were tumor and others.

**Table 1:** Demographic characteristics of the patients

Characteristics	Total (*n* = 95)	orthopedic surgical robot group(*n* = 44)	traditional fluoroscopy group(*n* = 51)	*p* value
Age-yr	57(54,68)	57(53.25,65)	59(54,71)	0.1579*
Female/male	67:28	30:14	37:14	0.6589&
BMI-kg/m^2^	23.22 ± 3.70	22.99 ± 3.30	23.43 ± 4.05	0.5681#

BMI: Body mass index

#: The values are given using t test; *: The values are given using Mann-Whitney U test; &: The values are given using Pearson χ² test

**Table 2:** Diagnosis of the patients

Diagnosis	Total (*n* = 95)	orthopedic surgical robot group(*n* = 44)	traditional fluoroscopy group(*n* = 51)	*p* value
Cervical fracture	46	24	22	0.3071&
Cervical spinal stenosis	32	13	19	0.5155&
Tumor and others	17	7	10	0.7898&

&: The values are given using Fisher’s exact test

**Fig. 2:**
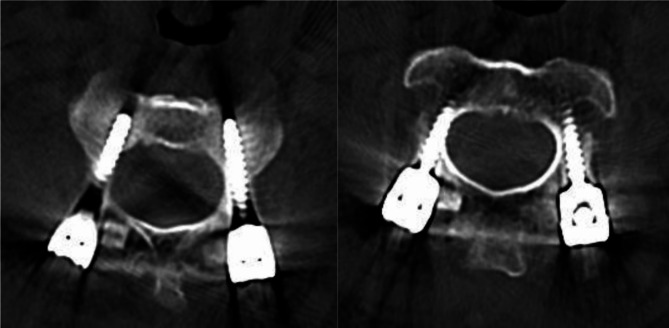
Postoperative CT image of the orthopedic surgical robot group A patient with atlantoaxial dislocation underwent open reduction and internal fixation of the cervical fracture using a robotic navigation system to implant pedicle screws at C1 and C2. Postoperative CT showed Neo Grade 1 at the right CPS of C2, and Neo Grade 0 at other screws

According to the Neo scale, 74.4% of all 422 screw placements were perfect (Grades 0) and 89.1% were acceptable (Grade 0 + Grade 1). In the orthopedic surgical robot group, 77.2% of 272 CPS were Grade 0, 15.1% Grade 1, 5.9% Grade 2, and 1.8% Grade 3, and the acceptable rate was 92.3% (Table 3; Fig. 2). The traditional fluoroscopy group showed that 69.3% of 150 CPS were Grade 0, 14.0% Grade 1, 6.7% Grade 2, and 10.0% Grade 3, and the acceptable rate was 83.3% (Table 4). Overall, compared with the traditional fluoroscopy group, the orthopedic surgical robot group had better accuracy in screw implantation that is, a higher acceptable rate of screws (*p* = 0.0083). Especially for pedicle screws placed in C1, C2, and C4 vertebrae, the acceptable rate of C1 vertebral screw placement was significantly higher than that of the traditional fluoroscopy group (*p* = 0.0195); for pedicle screws placed in C2 vertebrae, both its perfect rate (*p* = 0.0238) and acceptable rate (*p* = 0.0459) were significantly higher than those in the traditional fluoroscopy group; for pedicle screws placed in C4 vertebrae, its acceptable rate were significantly higher than that in the traditional fluoroscopy group(*p* = 0.018). There was no significant difference in the perfection rate or acceptance rate of other CPS.

**Table 3:** Neo scale of the orthopedic surgical robot group

orthopedic surgical robot group(*n* = 44)	Grade 0	Grade 1	Grade 2	Grade 3	Total	perfect rate (%)	acceptable rate (%)
C1	58	4	2	3	67	86.6(ns)	92.5(*)
C2	61	9	5	1	76	80.3(*)	92.1(*)
C3	19	8	1	0	28	67.9(ns)	96.4(ns)
C4	17	7	3	1	28	60.7(ns)	85.7(*)
C5	17	8	2	0	27	63.0(ns)	92.6(ns)
C6	15	3	0	0	18	83.3(ns)	100(ns)
C7	23	2	3	0	28	82.1(ns)	89.3(ns)
总计	210	41	16	5	272	77.2(ns)	92.3(**)

*: The significance of the differences in perfect rates or acceptable rates between Tables 3 and 4. The values are given using Fisher’s exact test. The asterisk (*) indicates *P* < 0.05. The double

asterisk (**) indicates *P* < 0.01

**Table 4:** Neo scale of the traditional fluoroscopy group

traditional fluoroscopy group(*n* = 51)	Grade 0	Grade 1	Grade 2	Grade 3	Total	perfect rate	acceptable rate
C1	7	0	0	4	11	63.6	63.6
C2	31	9	3	7	50	62.0	80.0
C3	3	2	0	0	5	60.0	100.0
C4	2	0	3	1	6	33.3	33.3
C5	6	0	0	0	6	100.0	100.0
C6	11	0	1	0	12	91.7	91.7
C7	44	10	3	3	60	73.3	90.0
总计	104	21	10	15	150	69.3	83.3

In addition, compared with the traditional fluoroscopy group, postoperative hospital stay was shorter in the orthopedic surgical robot group [7.432 ± 2.193 vs. 9.118 ± 5.102, *p* = 0.0447], but duration of surgery was longer [240(219,318) vs. 203(178,243) min, *p* = 0.0038], which may be related to more screws being implanted during surgery as well as robotic manipulation and intraoperative fluoroscopy procedures (Table 4). There was no significant difference in intraoperative blood loss between groups (*p* = 0.0872).

**Fig. 3:**
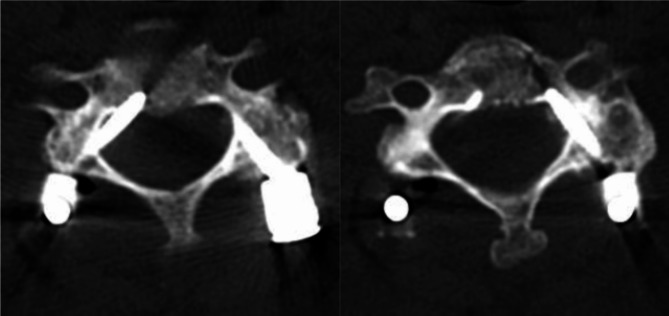
Postoperative CT image of the patient with decreased muscle strength in the traditional fluoroscopy group A patient with cervical malignancy underwent cervical spine tumor resection and pedicle screw fixation manually to implant pedicle screws at C4 and C5. Postoperative CT showed a 4.74 mm breach of the cortical bone at the right CPS and a 2.65 mm breach of the cortical bone at the left CPS of C4 vertebra. Neo scales of both sides of C5 were Grade 0. The patient presented with a decreased grip strength in the left hand, and refused to undergo a second operation. After 2 months of rehabilitation treatment, the patient’s left hand-grip strength was recovered from level 2 to level 4

In terms of postoperative complications, 2 cases of cerebrospinal fluid leakage and 1 case of decreased muscle strength occurred in the traditional fluoroscopy group. The 2 patients with cerebrospinal fluid leakage were released by continuous lumbar cistern drainage, suture of the drainage tube opening and other measures, and were discharged successfully after a few days. The patient with decreased muscle strength had a postoperative CT scan that showed a C4 right pedicle screw had entered the spinal canal, the Neo scale of which was Grade 3, and the left pedicle screw was Grade 2. The patient presented with a decreased grip strength in the left hand, and refused to undergo a second operation and chose to be discharged (Fig. 3). No infection or vertebral artery injury happened in the traditional fluoroscopy group and the orthopedic surgical robot group. There was 1 case of cerebrospinal fluid leakage in the orthopedic surgical robot group, but it was not related with orthopedic surgical robot navigation. (Table 5)

**Table 5:** Other outcomes

Variable	Total(*n* = 95)	orthopedic surgical robot group(*n* = 44)	traditional fluoroscopy group(*n* = 51)	*p* value
Duration of surgery -mins	228(182,290)	240(219,318)	203(178,243)	0.0038*
intraoperative blood loss -ml	319.8 ± 258.1	285.2 ± 274.4	349.6 ± 241.9	0.2273#
postoperative hospital stay -days	8.337 ± 4.094	7.432 ± 2.193	9.118 ± 5.102	0.0447#
Major complications -no./total (%)	4/95(4.21)	1/44(2.27)	3/51(5.88)	0.6211&

#: The values are given using t test; *: The values are given using Mann-Whitney U test; &: The values are given using Fisher’s exact test

## Discussion

Robotic technology has been used in manufacturing for decades, but it was only recently that it was applied to medicine, and it was even later that it received approval from the China Food and Drug Administration for clinical use in orthopedics. SpineAssist/Renaissance robot (Mazor Robotics, Caesarea, Israel) was firstly used for the spine, and some studies showed that screws that were implanted using the SpineAssist/Renaissance robot were successfully and accurately implanted [15, 16]. ROSA (Medtech, Montpellier, France) is another robot used in spinal surgery, and was reported to perform transforaminal lumbar interbody fusion (TLIF) accurately and safely [17]. 

Cervical spinal surgeon involving orthopedic surgical robotic assistance for CPS implantation may be associated with potential advantages relative to conventional traditional fluoroscopy-assisted CPS implantation. A meta-analysis involving 6 studies, including 2 controlled studies, showed a total of 482 cervical screws were placed with the use of a surgical robot, and 78.6% were CPS. 471 of 482 cervical screws (97.7%) achieved a clinically acceptable grade (a < 2-mm screw breach through the cortex) and yielded an average screw deviation of 0.95 mm [18]. 

Our study showed that the orthopedic surgical robot group implanted CPS more accurately than the traditional fluoroscopy group, with higher overall perfect rate and acceptable rate. It was worth noting that, the orthopedic surgical robot group performed particularly well in upper cervical vertebrae(C1-C2). Before our study, the Tianji robot had performed robot-assisted C1-C2 transarticular screw fixation for atlantoaxial instability and robot-assisted odontoid fracture fixation and got excellent results as the first reported clinical applications of robot-assisted cervical spinal surgery [10]. 

Compared with the traditional fluoroscopy group, postoperative hospital stay was significantly shorter in the orthopedic surgical robot group (*p* = 0.0447), which was consistent with a previous study by Fan et al. [19] Because the orthopedic surgical robot group had fewer complications, only one case of cerebrospinal fluid leakage (not related with orthopedic surgical robot), patients recovered faster and had shorter postoperative hospital stay. Cervical fractures are often associated with other injuries, which can significantly impact postoperative hospital stay. In this study, the proportion of cervical spine fractures between the two groups did not demonstrate a statistically significant difference. It is noteworthy that patients had previously undergone management for severe life-threatening injuries, including traumatic brain injuries, organ damage and others, before receiving cervical spine surgery. A review indicated that the average postoperative hospital stay for posterior cervical surgeries was 5.7 days [18]. In contrast, the average postoperative hospital stay in this study was 8.3 days, which was relatively prolonged. We believed this extended duration was primarily due to the higher proportion of cervical fracture, which often necessitate additional orthopedic interventions before discharge.

However, the duration of surgery of the orthopedic surgical robot group was significantly longer than the traditional fluoroscopy group(*p* = 0.0038). The increased surgical time could be partly attributed to the intraoperative preparation phase and additional intraoperative CT images of the patient are needed. Besides, the number of CPS inserted during surgery tended to be higher in the orthopedic surgical robot group (6.18 CPS per case) than in the traditional fluoroscopy group (2.91 CPS per case). This study focused solely on CPS. For safety reasons, when it was difficult to implant CPS, lateral mass screws were chosen instead, which was primarily the case in the traditional group. In addition to the duration of surgery, we believe that the number of pedicle screws may increase intraoperative blood loss (not significant in this study), but has no significant impact on postoperative hospital stay. When CPS was not suitable, lateral mass screws would be implanted, which had a minimal impact on surgical time and intraoperative blood loss, and no significant effect on postoperative hospital stay. In the total cost of the surgery, in addition to the higher expenses caused by more screws, the use of the Tianji robot incurs an additional charge of 27,000 RMB, which will be adjusted according to healthcare insurance policies.

In the present study, the intraoperative blood loss did not differ significantly between two groups. Likewise, in the study by Fan et al., the intraoperative blood loss in the orthopedic surgical robot group who underwent open surgery do not differ significantly from that of the traditional fluoroscopy group [19]. Nevertheless, a few studies showed that the robot-assisted minimally invasive percutaneous cervical spinal surgery led to significantly less intraoperative blood loss, and this can be a huge advantage of the orthopedic surgical robot [19, 20]. Besides, the incidence of postoperative complications was similar between two groups (*p* = 0.6211).

In the meta-analysis, after excluding the studies involving other types of cervical screws, the acceptable rate of CPS was 96.9% [18]. Factors contributing to deviation in robot-assisted surgery include slippage on the bone surfaces of the entry point, surgeon’s surgical technique, marker displacement, respiratory amplitude and muscle stretching [21–24]. Compared with the reported meta-analysis of robot-assisted CPS fixation, our study had a lower acceptable rate (92.3% vs. 96.9%) [18]. Further reason may be due to the lateral deviation caused by muscle traction during the open surgical procedure. It has been reported that the use of percutaneous needle placement may reduce the effects of muscle traction and improve nail placement accuracy [24]. However, our study had a larger sample size (number of CPS) than other studies, so it has certain persuasion.

Several limitations existed in our study. First, this study was a single-center study, so a multi-center study is needed for more convincing results. Second, although the study showed improved accuracy for screw implantation in the orthopedic surgical robot group, long-term follow-up is needed to confirm the better prognosis. Third, although the total number of CPS was relatively enough, the pedicle screws implanted in C3 and C4 were relatively few.

In conclusion, this retrospective study showed that the accuracy of spine surgery with CPS implantation using orthopedic surgical robot-assisted technique tended to be superior to traditional fluoroscopy-assisted technique, while maintaining comparable safety at the same time.

## Data Availability

All data and materials are available by contacting the corresponding author.
